# Quantifying dimensions of physical behavior in chronic pain conditions

**DOI:** 10.1186/s12984-016-0194-x

**Published:** 2016-09-23

**Authors:** Anisoara Paraschiv-Ionescu, Christophe Perruchoud, Blaise Rutschmann, Eric Buchser, Kamiar Aminian

**Affiliations:** 1 Laboratory of Movement Analysis and Measurement, STI, Station 11, Ecole Polytechnique Federale de Lausanne (EPFL), CH1015 Lausanne, Switzerland; 2 Anesthesia and Pain Management Department, EHC, Hospital of Morges, Morges, Switzerland; 3 Department of Anesthesiology, University Hospital Center and University of Lausanne, Lausanne, Switzerland

**Keywords:** Chronic pain assessment, Physical behavior, Pattern complexity, Factor analysis, Composite scores

## Abstract

**Background:**

Chronic pain, defined as persistent or recurrent pain lasting longer than 3 months, is a frequent condition affecting an important percent of population worldwide. Pain chronicity can be caused by many different factors and is a frequent component of many neurological disorders. An important aspect for clinical assessment and design of effective treatment and/or rehabilitation strategies is to better understand the impact of pain on domains of functioning in everyday life. The aim of this study was to identify the objectively quantifiable features of physical functioning in daily life and to evaluate their effectiveness to differentiate behavior among subjects with different pain conditions.

**Method:**

Body worn sensors were used to record movement data during five consecutive days in 92 subjects. Sensor data were processed to characterize the *physical behavior* in terms of type, intensity, duration and temporal pattern of activities, postures and movements performed by subjects in daily life. Metrics quantifying these features were subsequently used to devise composite scores using a factor analysis approach. The severity of clinical condition was assessed using a rating of usual pain intensity on a 10-cm visual analog scale. The relationship between pain intensity and the estimated metrics/composite scores was assessed using multiple regression and discriminant analysis.

**Results:**

According to the factor analysis solution, two composite scores were identified, one integrating the metrics quantifying the amount and duration of activity periods, and the other the metrics quantifying *complexity* of temporal patterns, i.e., the diversity of body movements and activities, and the manner in which they are organized throughout time. All estimated metrics and composite scores were significantly different between groups of subjects with clinically different pain levels. Moreover, analysis revealed that pain intensity seemed to have a more significant impact on the overall physical behavior, as it was quantified by a global composite score, whereas the type of chronic pain appeared to influence mostly the complexity of the temporal pattern.

**Conclusion:**

The methodology described could be informative for the design of objective outcome measures in chronic pain management/rehabilitation programs.

## Background

Chronic pain is a complex disabling experience affecting at least 10 % of the world’s population, with an estimated prevalence closer to 20–25 % in some countries and regions [1]. The high prevalence is due to the many factors contributing to the development and persistence of pain, including degenerative and inflammatory diseases, nerve injury and neurological conditions (Parkinson’s disease, stroke, multiple sclerosis) among many other factors [2, 3]. The most important features of chronic pain experience are *pain severity* (intensity), *pain interference* (with work performance, participation in recreational activities, ability to perform activities of daily living, social activity) and *emotional distress* (depression, anxiety, coping behaviors) [4]. These factors may influence physical behavior in various and intricate ways: stop an activity because of increasing pain or, on the contrary, persist exaggeratedly with tasks despite severe pain due to maladaptive coping strategies; avoid painful body movements and specific activities due to emotional distress or on the contrary, break a task into pieces and go slower [5–8]. Although the patterns of functioning play a central role in models of chronic pain and disability and in evaluation of treatment/rehabilitation efficacy [4, 7, 9–24], the relationships between clinical conditions of patients and their behavior in the context of everyday life remain difficult to be characterized comprehensively. The assessment methods need to take into account the *multidimensional*, *dynamic* and *relational* attributes of physical behavior. The *multidimensional* attribute refers to the many characteristic features, i.e., *type*, *intensity* and *duration* of various activities, movements and postures, whereas the *dynamic* aspect refers to their continuous change over time that give rise to *temporal patterns*. The *relational* aspect refers to the many factors that may modulate the behavioral pattern of the person, i.e., biological, environmental, lifestyle, etc.

The aim of this study was to explore the potential of wearable technology combined with appropriate analytical methods for data analysis, to comprehensively characterize the individuals’ physical behavior in everyday life. Specifically, the objectives were to: *(1)* devise a set of metrics to quantify multiple dimensions of daily functioning, given that individuals with different pain levels may, for example, accumulate the same time percent of walking but with episodes of different duration and intensity in terms of speed/cadence; *(2)* integrate multiple metrics into composite scores, supposed to provide a more complete assessment of the physical functioning; *(3)* evaluate the relationship between a rating of usual pain intensity and the estimated metrics/composite scores. Overall, the study aims to illustrate that an objective and detailed characterization of physical behavior in daily life, may open new perspectives for outcome evaluation in future clinical intervention studies.

## Methods

### Subjects and study design

The analysis was conducted retrospectively on a dataset that included movement registration data in 92 subjects, including 74 chronic pain patients and 18 pain-free individuals. Patients were referred to the Pain Management Center of the Hospital of Morges, Switzerland, because of long-lasting persistent intractable pain and were candidate for spinal cord stimulation therapy. Pain-free subjects were volunteers recruited from the patients’ relatives or the medical staff of the clinic. After the approval of the local ethical committee (University of Lausanne, Switzerland) and written informed consent was obtained from each participant, body movements were recorded under free-living conditions, during five consecutive weekdays, 8 h each day. Patient recordings were obtained before the spinal cord stimulation treatment. Data collected were body accelerations, recorded 40 samples per second with custom-made (non-commercial) data-logger devices including commercial sensors (tri-axial accelerometer, MMA7341LT, range ±3 g, Freescale, Austin, TX, USA), a battery (3.7 V, 595 mA/h), a memory unit and a microcontroller. Devices were small and lightweight (55 × 40 × 18 mm, 50 g) and were stuck to the skin with medical adhesive patches; one on the sternum to measure the trunk accelerations and one on the mediolateral axis of the thigh. Subjects were instructed to install devices and start recording in the morning before engaging in daily activities.

Chronic pain condition was assessed using a global rating of usual pain intensity experienced by subjects during the monitoring period. Each participant was asked to rate his/her perceived pain on a 10-cm visual analogue scale (VAS) from 0-cm (no pain) to 10-cm (worst imaginable pain). Table 1 shows the subjects’ demographic data.

**Table 1:** Subjects demographic data

Age (yrs)	63 ± 14
BMI (kg/m^2^)	26 ± 5
Gender, *n* males (%)	44 (47 %)
Employed, *n* (%)	40 (43 %)
Diagnosis, *n* (type)	22(SS), 25(FBSS), 10(CRPS), 8(PAD), 9(CP), 18(healthy/pain free)

*SS* spinal stenosis (*n*=, FBSS = failed back surgery syndrome, *CRPS* Complex regional pain syndrome, *PAD* peripheral artery disease, *CP* combined pathologies: herniated disc (*n* = 3), polyneuropathies (*n* = 3), deafferentation (*n* = 2), meralgya (*n* = 1)

### Metrics to quantify dimensions of physical behavior

The recorded trunk and thigh acceleration data were processed by validated software routines [25, 26] to classify body postures (sitting, standing, lying) and walking activity, to calculate the number of steps and cadence of walking periods, and to quantify intensity of movements in terms of peak body accelerations. The aggregated periods of standing and walking, indicating the time spent on feet, were defined as *activity* and, sitting and lying as *sedentary *(diurnal rest).

The time percentage spent walking and on feet (metrics *walking (%), activity (%)*) were selected to represent the *amount/volume* of physical activities [27, 28]. Given that similar time percentages might be obtained with many short periods, or a few long, or a combination of both, it appears meaningful to characterize also the *duration of periods*. Empirical evidence indicates that in real-life conditions *activity* periods may range from seconds to minutes and hours, therefore the standard central tendency statistics (mean, median) are not appropriate measures. One alternative is to characterize maximal values presumed to reflect better the functional capacity of the subject. The simpler statistical measures are the sample extreme upper quantiles, which are more robust to outliers compared with single maxima value. For the ensemble of *activity* periods performed by a subject, we selected the .975^th^ quantile who returned a value indicating that 2.5 % of *activity* periods were longer than that value (metric *q.975 actv.*).

Further information was deduced from the relationship between successive *activity* and *sedentary* periods, i.e. by assessing if and to what extent a subject needed longer rest after an activity period. The assumption was that the duration of sedentary or resting time after a physically demanding (and potentially painful) activity period may increase in chronic disease conditions. Two sets of values denoted ‘*excess rest*’ and ‘*deficit rest*’ were estimated: ‘excess rest’ was associated to the amount (in seconds) by which a *sedentary* period was *longer* when compared with the preceding *activity* period; conversely, ‘deficit rest’ was associated to the amount by which a *sedentary* period was *shorter* when compared with the preceding *activity* period. The idea was to plot on the same diagram the empirical cumulative distribution functions (ECDF) of the two sets of values (‘excess’ and ‘deficit’) and to estimate the statistical distance between the curves using the signed Kolmogorov-Smirnov (KS) distance (metric *KS dist.*). The KS test was conducted to test the null hypothesis that the two ECDFs were equal against the alternative hypothesis that the ECDF of deficit rest is larger than ECDF of excess rest (i.e., the ECDF plot of deficit rest is above the ECDF plot of excess rest, so *higher values of KS distance indicates tendency of longer rest after activity*).

Along with the amount and duration of periods, an additional dimension of physical behavior is the temporal sequence/pattern of various states, where the states describe various movement features, for example, walking periods with various durations and cadences, sitting/standing quiet or with body movements, transitions between movements and activities [29]. Patterns containing a higher diversity of states, changing dynamically over time, are considered more ‘complex’ and are associated with better physical capability [29]. Entropy, as a fundamental measure of complexity, was used under different formulations in order to quantify: *(i)* the diversity of physical activities states (entropy 1); *(ii)* the diversity and temporal dynamics of states, i.e., moment-to-moment variations of activities and movement features (entropy 2, entropy 3) [29, 30]. Higher entropy/complexity values were associated with better physical functioning, i.e., the ability to perform a wide range of movements and actions and to timely respond to environmental demands.

All metrics were estimated from aggregated data obtained from complete recordings of five consecutive days.

### Exploratory factor analysis to determine composite scores

The associations between metrics and possibility to combine related ones to define composite scores were investigated using exploratory factor analysis (EFA). The analysis was conducted according to recommendations for best practice in EFA [31–33], and started with validation of the basic assumptions for data adequacy. The first assumption concerned factorability of correlation matrix, i.e., the presence of appropriate linear correlations among metrics so that coherent composite scores can be identified. According to recommendations, the pairwise correlations coefficients should be in the range 0.8 ≥ |*r*| ≥ 0.4, which means that *metrics should correlate but not too high*, to avoid multicollinearity and singularity, which reduce the clarity of EFA solution. The second assumption was adequacy of sample size to yield reliable estimates of correlations among metrics. Ideally, there should be a large ratio of *N/k*, where *N* is the number of subjects and *k* the number of metrics (minimum 5 subjects per metric required). According to the literature, normal distributed data enhances EFA solution but is not a strict assumption if appropriate method is used for factor extraction [33].

After confirmation of data adequacy, all metrics were standardized by subtracting the mean and dividing by sample standard deviation to accommodate the different scales (z-scores). One metric (*KS dist.*) was multiplied by -1 so that all metrics were positively correlated, with increasing values associated with increasing physical activities. The set of metrics was then submitted to a principal axis factoring recommended for non-normal data, with oblique rotation (promax) to extract the *factors* (linear combinations of interrelated metrics). The optimal number of factors was determined according to scree plot criteria and parallel analysis method [32, 33].

The EFA procedure provides two types of results, the *factor loadings* and the *factor scores*. In the context of our analysis, the *factor loadings* represent the relationship of each metric with the underlying factor. The degree of association between metrics and factors was evaluated according to a minimal factor loading threshold (≥0.3) and the requirement of at least three metrics with high loadings per factor. The factors were labelled according to the consistency among the metrics that loaded high on each factor.

The *factor scores* were composite measures (standardized to z-scores) created for each subject on each factor [34]. Generally, two approaches can be used to derive subjects’ composite scores [35]: (1) *weighted scores,* calculated as weighted summation of standardized metrics associated to each factor and (2) *unit-weighted scores,* calculated as simple summation of standardized metrics associated to each factor, so that each metric on the factor contribute equally to the composite. The composite scores were used as outcome measures in subsequent analysis aiming to evaluate the relationship between pain intensity and overall physical behavior.

### Association between pain intensity and physical behavior

The relationship between intensity of pain evaluated on the VAS, and physical behavior quantified with the set of metrics/composite scores, was assessed using multiple regression and discriminant analysis. Multiple linear regression analysis was conducted with the metrics/composite scores as dependent variables, and VAS score, age and BMI as independent variables. Discriminant analysis was performed to determine how significantly the metrics/composite scores were different between groups of subjects with clinically different pain intensities (age-matched). For this purpose, the 92 subjects were divided into two groups according to pain intensity: the first group (*n* = 34) included subjects with VAS score inferior or equal to 4 (*mild pain*) and the second group (*n* = 58) included subjects with VAS superior to 4 (*moderate to severe pain*). Based on normality test (Shapiro-Wilk) the differences between groups were assessed using two-sided Student’s t-test or nonparametric Mann-Whithney test. The effect size (magnitude of the difference) was estimated using the Cohen’s *d* and the corresponding percent of non-overlap between groups [36]. The whole analysis was performed using MATLAB computing software (version R2013a, MathWorks, Natick, MA, US).

## Results

### Quantified physical behavior and differences among individuals

**Fig. 1:**
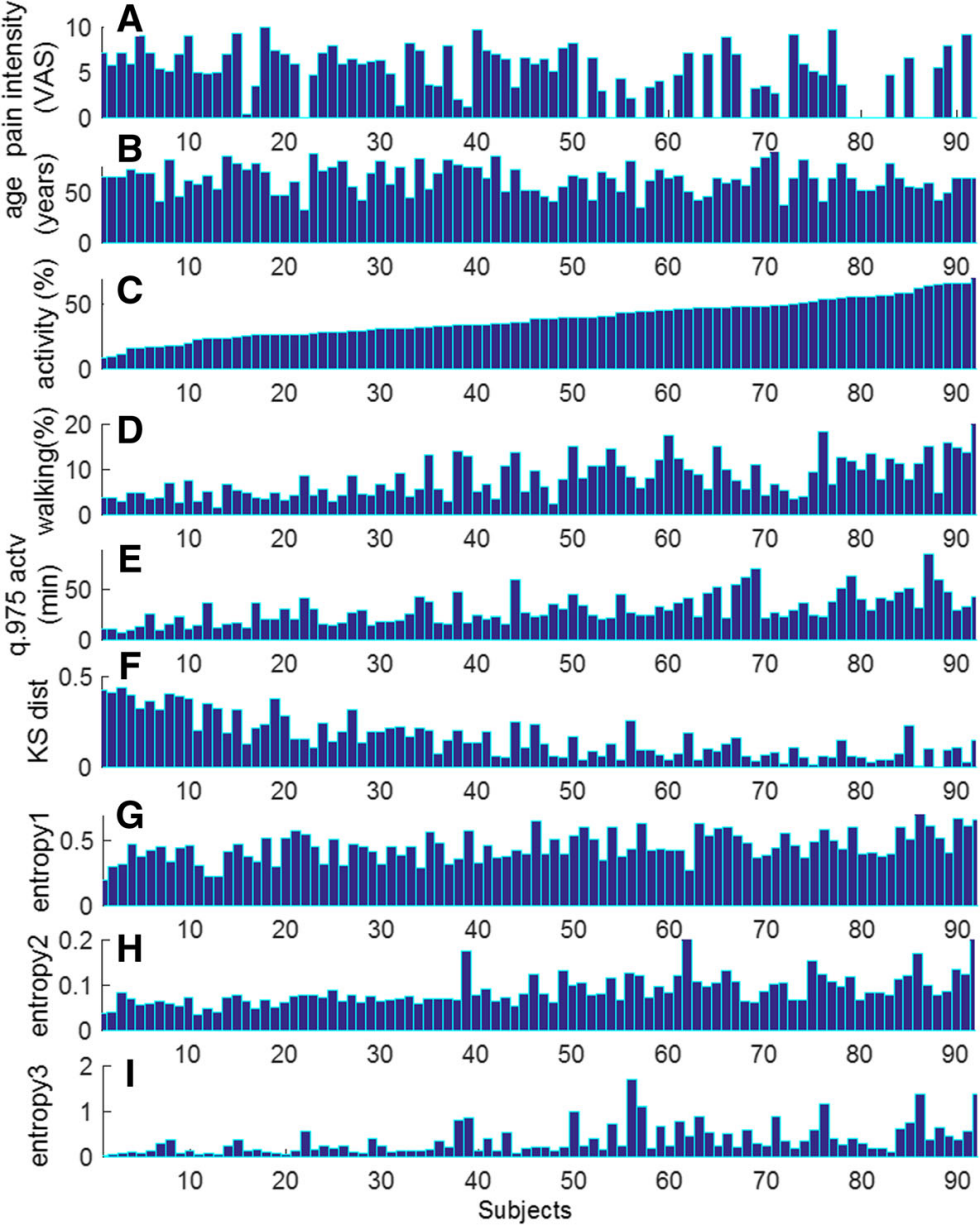
Pain intensity (**a**), age (**b**) and PA metrics (**c**-**i**) estimated for each subject (*N* = 92). This representation highlights variability of physical behaviors in every-day life, and how a similar amount of *activity*, expressed as percentage over monitoring time (**c**), is accumulated from patterns characterized by different amount of walking (**d**), different duration of activity periods (**e**), different duration of sedentary periods following activity (**f**) and different complexity of temporal patterns (**g**, **h**, **i**)

Self-reported pain intensity (VAS score), age and the set of metrics estimated for each subject are illustrated in Fig. 1a-i. These results suggest that variability of behaviors among individuals may result from the fact that the various components of everyday life physical activities are affected in different ways by clinical condition of the subjects. For illustrative purposes, Fig. 1c shows the percent of time spent in *activity* (on feet), which is a usual metric, for the ensemble of subjects (*N* = 92) as they score from low to high values. These representations indicate that some subjects with different pain intensities (Fig. 1a) and ages (Fig. 1b) have accumulated similar amount of *activity* during the five monitoring days; however, differences were revealed by the amount of locomotion during the time on feet (Fig. 1d, *walking* (%)), duration and succession of *activity* and *sedentary* periods (Fig. 1e, f, *q975.actv., KS dist.*), and by *intensity* and *diversity* of physical activities (Fig. 1g, h, i, *entropy* metrics).

**Fig. 2:**
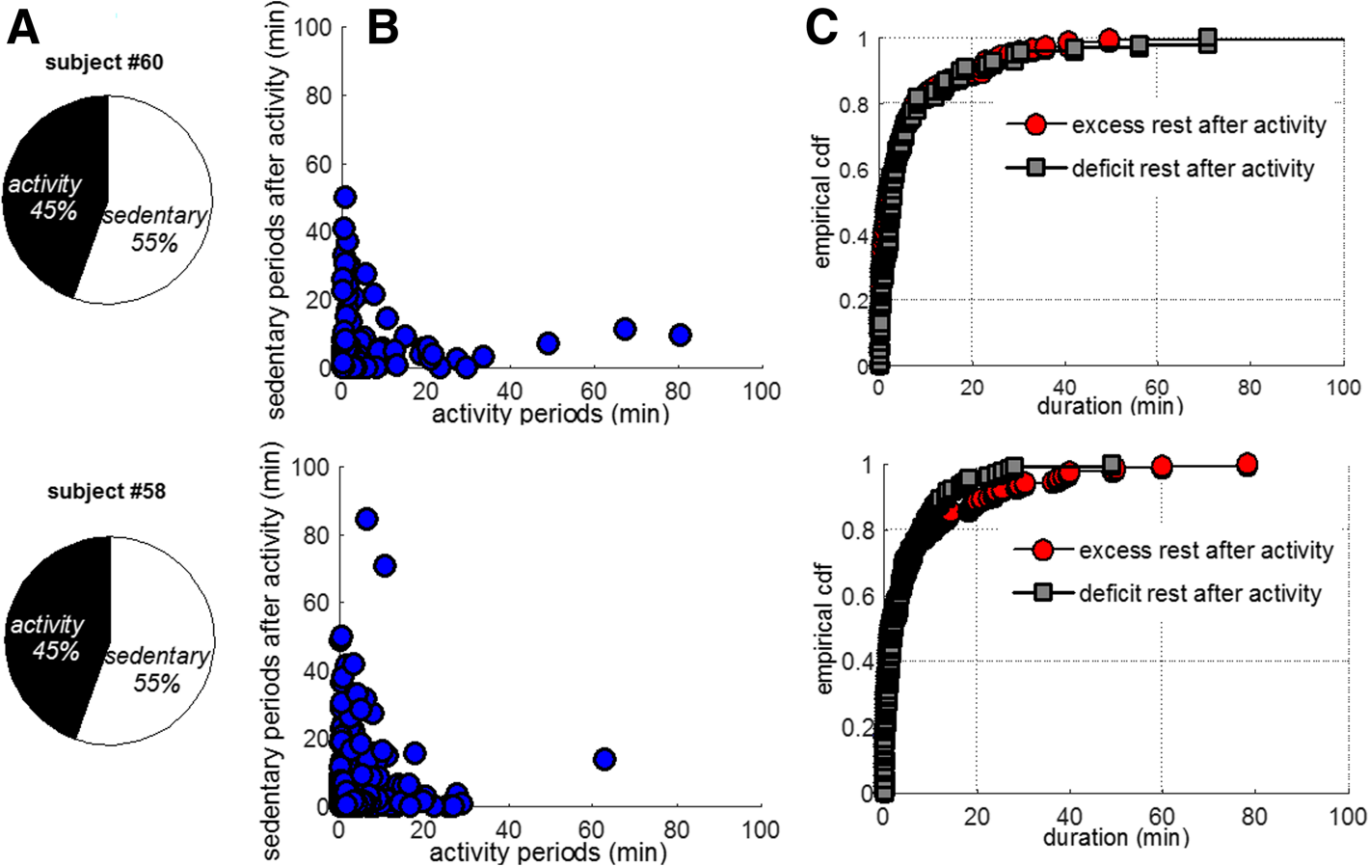
Comparative illustration of metrics quantifying aspects of physical behavior in two subjects with different pain condition (matched by age): although the total time spent in activity/sedentary was similar (**a**), differences were noticed in the duration of respective periods (**b**), as well as in the duration of sedentary time after activity (**c**)

**Fig. 3:**
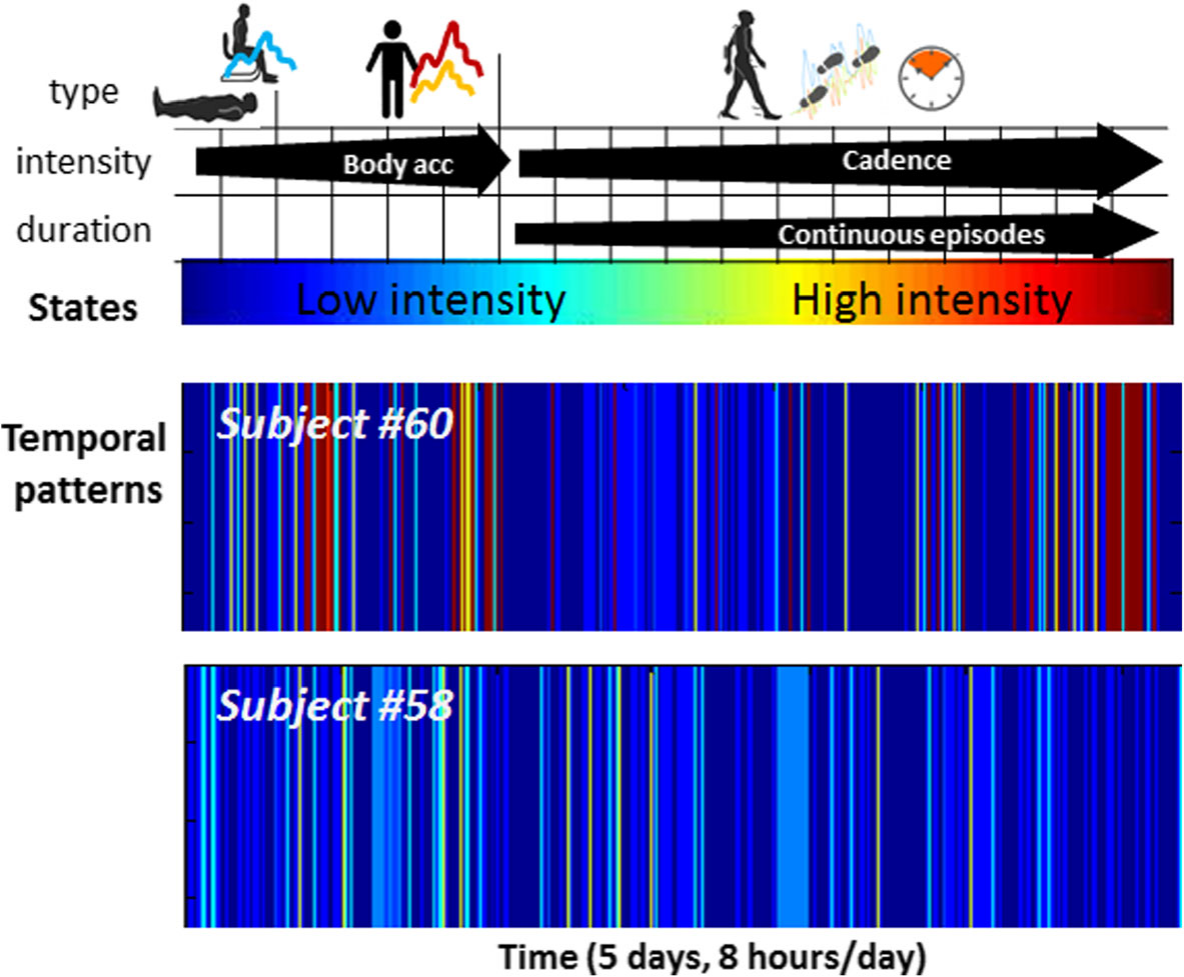
Definition and visualization of temporal patterns: the information about *type, intensity* and *duration* is integrated into several states (here a number of 18). Low intensity states (‘*cold*’ colors) are associated with sedentary postures whereas higher intensity states (‘*warm*’ colors) integrate the standing posture with various body accelerations (e.g. daily tasks, exercises) and walking periods characterized by various durations and cadences [29]. Visualization of these patterns provides an overview of the subject’s physical behavior during the monitoring period

For better illustration, Fig. 2 depicts metrics characterizing aspects of physical behavior of two male subjects with different pain intensities (Subject #60: VAS = 0, age = 64 years, and Subject #58: VAS = 4, age = 62 years). Both have spent a similar amount of time in *activity* (Fig. 2a). However, subject #60 performed a few longer periods and many of his *activity* periods were followed by shorter or similar *sedentary* periods, while subject #58 performed longer *sedentary* periods and his *activity* was much more fragmented (scatter plot in Fig. 2b). The ECDF plots shown in Fig. 2c and the KS statistical distance (defined as maximum vertical deviation between curves) indicate for the chronic pain patient a tendency to prolong systematically *sedentary* time after *activity *(increased *KS dist.*). Additional differences were revealed by the temporal patterns (‘barcodes’) that include detailed information about the type (lying/sitting, standing, walking), intensity (body acceleration, walking cadence), duration (continuous walking) and frequency of various activities and body movements. The patterns illustrated in Fig. 3 are characterized by relatively similar dynamics (moment-to-moment changes between states) however it can be observed that the pattern of subject #60 contains a higher *diversity* of states, ranging from low intensity (blue color) to high intensity (red color) and therefore appears more *complex* (i.e., characterized by higher entropy values).

### Exploratory factor analysis: identified composite scores

**Fig. 4:**
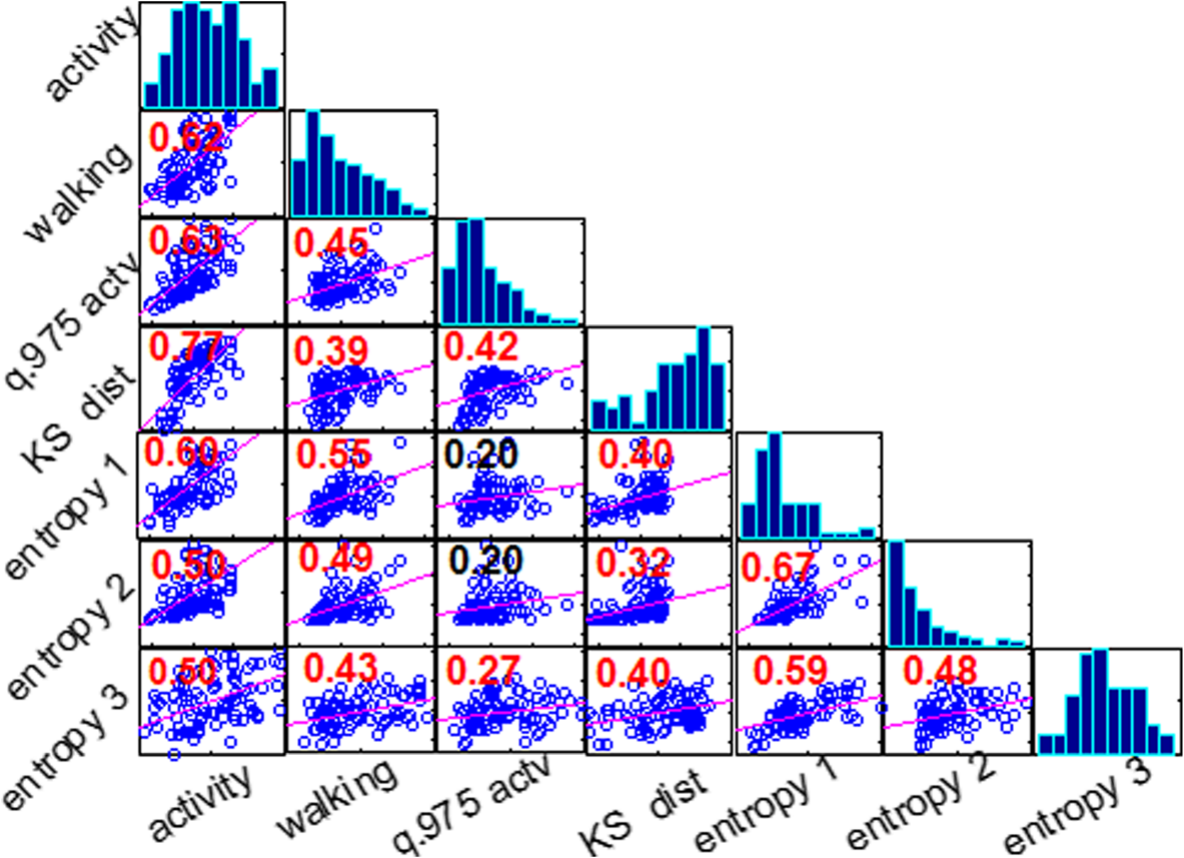
Matrix of plots showing correlations among pairs of metrics estimated for each subject (*N* = 92): histograms of metrics appear along the matrix diagonal and pairwise relationships between metrics (scatter plots) appear off diagonal. The slopes of least-squares reference lines in the scatter plots are equal to the displayed correlation coefficients (*red color* number if statistically significant)

The correlation matrix indicated linear relationships and appropriate pairwise correlations between metrics (Fig. 4). The sample size requirements were also satisfied, since the study included *N* = 92 subjects and *k* = 7 metrics (ratio approx. 13 to 1). Cattell’s scree test and parallel analysis indicated an optimal two factors solution accounting for 60 % of the variance among metrics. The factor loadings for the set of metrics, the proportion of variance explained by each factor, and communality (i.e., the proportion of the variance of each metric explained by the two common factors) are presented in Table 2. The factor loadings were examined to determine metrics that have the strongest correlations with a given factor, and thus to provide an interpretation for that factor. Metrics a*ctivity(%)*, *walking(%)*, *q.975 actv.*, and *KS dist.* appeared highly loaded on Factor 1, so, this factor was named ‘*Mobility*’; the *entropy* metrics were strongly loaded on Factor 2, therefore, it was named ‘*Complexity*’. All weights were positive suggesting a direct correlation among metrics within each factor.

**Table 2:** Factor loadings for the two-factor EFA solution

PA metrics	Factor loadings		Communality
	Factor 1 ‘*Mobility*’	Factor 2 ‘*Complexity*’	
*Activity (%)*	**0.91**	0.12	0.85
*Walking (%)*	**0.40**	0.34	0.3
*q.975 actv. (min)*	**0.77**	−0.19	0.64
*KS dist.*	**0.78**	−0.01	0.6
*Entropy 1*	0.01	**0.72**	0.52
*Entropy 2*	−0.04	**0.96**	0.92
*Entropy 3*	0.11	**0.56**	0.33
% of variance explained	31 %	29 %	

The metrics highly associated with each factor are represented in bold characters

**Fig. 5:**
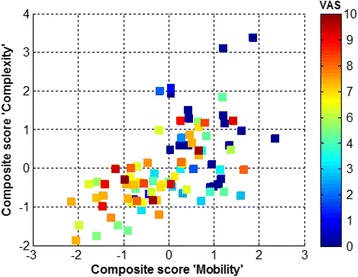
Scatter plot of composite scores, labelled *Mobility* and *Complexity*, for the ensemble of subjects; the color bar encodes subjects’ pain intensity, from 0 (*in blue*), to maximum value of 10 (*in red*). This representation indicates that: (1) there is a positive relationship between the two scores (correlation coefficient *r* = 0.58, *p* < 0.0001); (2) a number of 39 subjects with high pain intensity (VAS = 6.2 ± 2) have negative values for both scores (bottom left quadrant), i.e., values under the mean value of the entire sample of 92 subjects

Weighted and unit-weighted factors defining the composite scores were derived for each subject using the results in Table 2. The literature [35] suggests that the unit-weighted approach is more robust for subsequent analysis (or replicated studies) because it is less influenced by deviations from normality or outliers in the original data (i.e. the set of metrics estimated from the sample of subjects). Taking into consideration this recommendation and the strong correlation found between the composite scores obtained with the two approaches (*r* > 0.9), the unit-weighted composite scores were retained for subsequent assessment. A global composite score was also created as the sum of unit-weighted *Mobility* and *Complexity*, given that they were themselves correlated (*r* = 0.58, *p* < 0.001) (Fig. 5), and theoretically related.

### Relationship between pain intensity and physical behavior

Multiple regression analysis revealed significant negative association between pain intensity (VAS) and the global composite score (standardized *Beta* = -0.15, *p* < 0.001) when controlling for age (*Beta* = -0.009, n.s.) and BMI (*Beta* = -0.001, n.s.). The overall model fit has been found to be *R* ^*2*^ = 0.25 (F-statistic for change in *R* ^*2*^: 14.6, *p* < 0.001) indicating that 25 % of variance in the global scores was explained by pain severity. No significant relationship was found between pain intensity and the metrics quantifying various aspects of physical behavior (Fig. 1c-i).

**Fig. 6:**
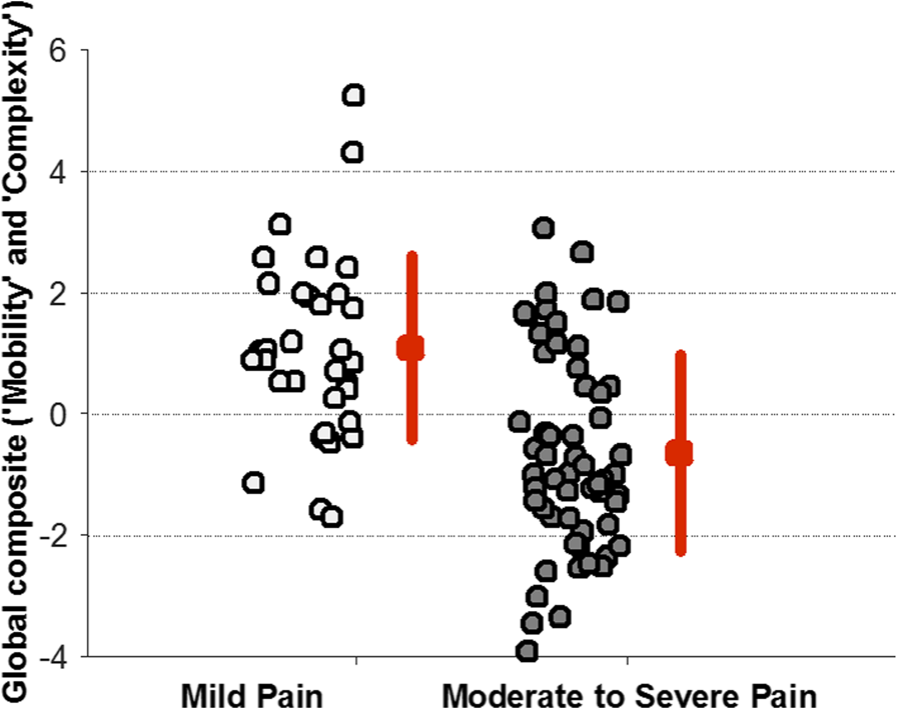
Values of the global composite score for groups of subjects with clinically different pain intensity. The graph shows the group mean and standart deviation as well as values corresponding to each subject. Estimation of the effect size (Cohen’s d) indicates approximativelly 60 % nonoverlap between groups

Table 3 contains the averaged values of metrics/composite scores (mean ± std) estimated for two groups of subjects with clinically different pain levels (matched by age). According to Mann-Whithney test and Cohen’s *d* statistic, all metrics/composite scores were significantly different between groups and were characterized by large effect size. The best discriminant property was observed for the global composite score characterized by the largest effect size, corresponding to approximately 60 % non-overlap between groups (Fig. 6).

**Table 3:** Statistical significance of differences between the two groups of subjects

Clinical variables & PA metrics	Mild pain (*n* = 34)	Moderate to severe pain (*n* = 58)	p-val	Effect size: Cohen’s d (% of non-overlap between groups)
*Pain intensity (VAS)*	1.3 ± 1.5	6.8 ± 1.4	1.2e-15	3.60 (100 %)
*Age(yrs)*	64 ± 14	62 ± 13	0.47	0.13 (8 %)
*Activity (%)*	41 ± 17	34.5 ± 14	0.0009	0.78 (~47.1 %)
*Walking (%)*	9.5 ± 6	6.5 ± 4	0.001	0.75 (~45.4 %)
*q.975 actv. (min)*	37 ± 17	26 ± 12	0.002	0.73 (~44 %)
*KS dist.*	0.1 ± 0.07	0.2 ± 0.12	0.0007	0.84 (~48 %)
*Entropy 1*	0.52 ± 0.11	0.42 ± 0.10	0.001	0.79 (~47.2)
*Entropy 2*	0.045 ± 0.02	0.03 ± 0.01	0.0004	0.82 (~47.8)
*Entropy 3*	0.51 ± 0.40	0.28 ± 0.20	0.001	0.80 (47.4 %)
*Composite score ‘Mobility’*	0.62 ± 0.73	−0.36 ± 0.96	0.0004	1.10 (58.9 %)
*Composite score ‘Complexity’*	0.45 ± 0.9	−0.26 ± 0.71	0.0007	0.81 (~47.5 %)
*Global composite score* *‘Mobility & Complexity’*	1.08 ± 1.5	−0.63 ± 1.60	1.1e-5	1.15 (~60 %)

### Variation of physical behavior among subgroups of patients with different diagnostic

**Fig. 7:**
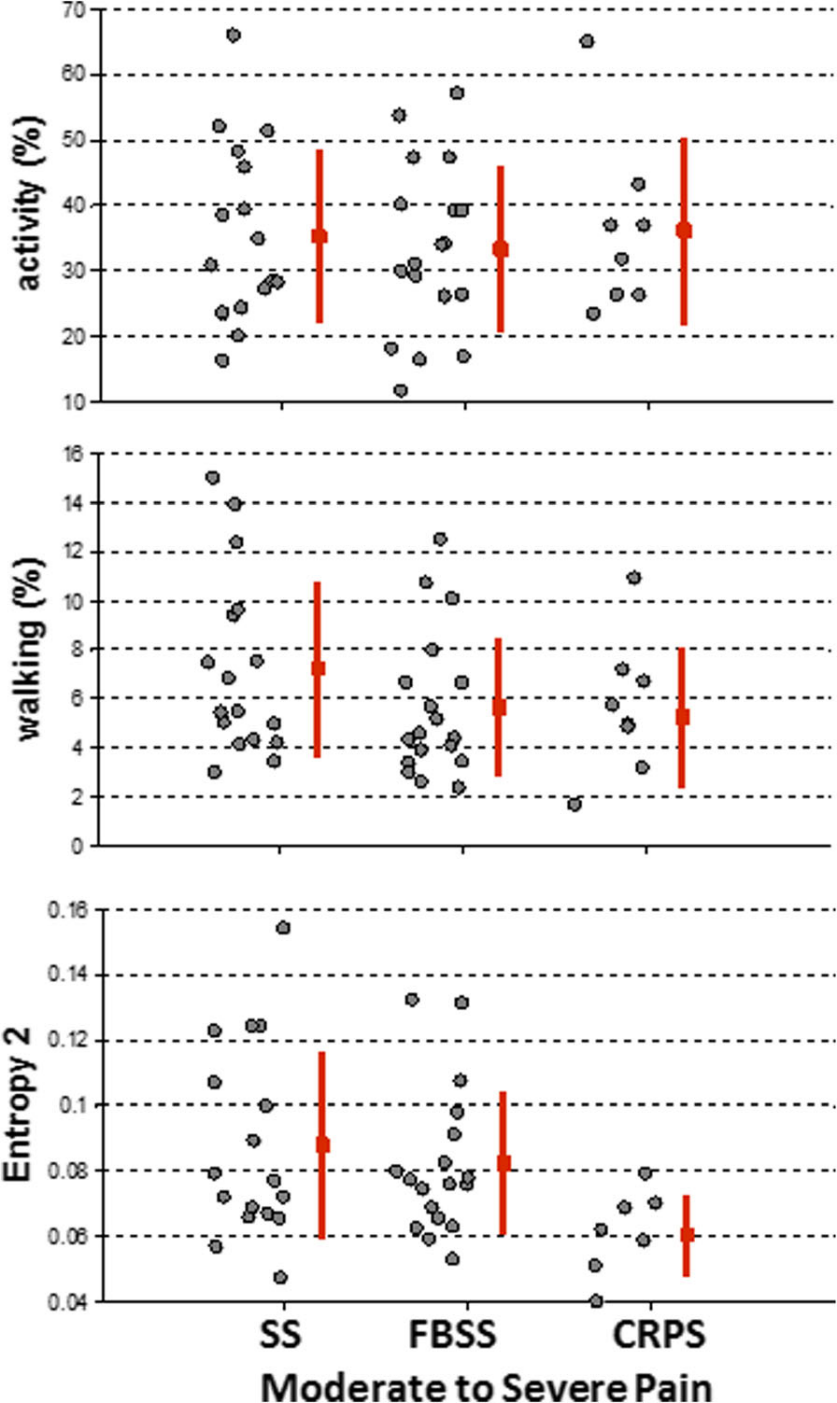
Variation of metrics between groups of subjects with chronic pain caused by spinal stenosis (SS), failed back surgery syndrome (FBSS) and complex regional pain syndrome (CRPS). Lower entropy values for CRPS patients indicate a reduced diversity of body movements/activities, low movement intensity and long sedentary periods, suggesting the potential of this metric to capture clinically recognized motor-impairements

The large within-group variability observed for all metrics has led to the hypothesis that, in addition to pain severity, another influencing factor of physical behavior was the *type* of pain. P*ost-hoc* analysis was conducted on the available dataset to compare the metrics of patients with *similar pain intensity* (moderate to severe pain) but different *diagnosis,* as follows: spinal stenosis (SS, *n* = 18, VAS = 6.8 ± 1.6, age = 72 ± 10), failed back surgery syndrome (FBSS, *n* = 19, VAS = 7 ± 1.5, age = 57 ± 12), and complex regional pain syndrome (CRPS, *n* = 7, VAS = 6.4 ± 1.4, age = 53 ± 13). Criteria for selection were the homogeneity of diagnostic within each group and similar pain intensity between groups. Figure 7 illustrates two conventional metrics, *activity* (%) and *walking* (%), and *complexity* of temporal pattern (entropy 2). These representations indicate no difference between the three groups for *activity* or *walking*, however lower *entropy* values seems to differentiate CRPS group from SS and FBSS groups. Although the sample size was small and this observation needs additional validation in future prospective studies, it appears consistent with the clinical description of motor impairments in CRPS, i.e., ‘*bradykinesia, deficit in movement amplitude, reduced frequency of movements*’ [37]. The sequence of states defining the temporal patterns/barcodes includes information related to all these movement features, so, this may explain why the *entropy* metric was lower and more discriminative for CRPS patients.

## Discussion

Technological developments during the last decade have made available various solutions for physical activity monitoring, allowing collection of data over long periods in the natural environment of the subject. However, an important step in the path from raw data to clinical evidence is the extraction of relevant information in terms of metrics characterizing the dimensions of physical behavior that give rise to distinctive patterns of functioning in everyday life. Analysis of data collected during five consecutive days in 92 subjects provides empirical evidence that ongoing pain may affect numerous aspects of physical functioning in everyday life. Changes appear to occur with respect to various features of physical behavior, from the overall amount of activity, to the duration of periods, and the complexity of the temporal pattern, characterized by ability to span a wide range of movements/activities within a given timeframe (Figs. 1, 2 and 3, Table 3).

### *Complexity* as a defining feature of healthy status: significance for assessment of chronic pain conditions

The concept of complexity emerged two decades ago in physiological research and it is now generally accepted that healthy physiological processes are ‘complex’ in that they are composed of fluctuations with information-rich structure [38, 39]. The structural richness characterizes the capacity of healthy physiological function to detect, respond and adapt to the innumerable perturbations and stressors of everyday life. The concept was advanced to postulate that disease and aging process could be defined by a progressive loss of complexity within the dynamics of physiologic outputs [40]. This was shown in a number of diseases and syndromes affecting cardiovascular, respiratory, central nervous and motor control systems [41].

Similar to physiological behavior, it is assumed that physical behavior generates an ‘output’ that can be modelled as a temporal pattern. Highly complex patterns (high entropy) are supposed to reflect healthy status and high level of functioning because results from freedom of movement and ability to perform daily tasks, physical performance, diversity of activities and participation in social life. Chronic diseases (long-lasting pain, fatigue, depression) may lead to progressive movement impairment, difficulties with daily tasks, limitation or avoidance of some activities i.e., a less complex (low entropy) pattern [29, 42]. The relevance of this concept in the context of chronic pain assessment is supported by the significant decrease of complexity metrics observed for the group of subjects with moderate to severe pain intensities, and more significantly for the patients with CRPS.

### Multidimensional quantification of physical behavior: implications for clinical assessment

The results in Table 3 indicate that pain intensity has a negative impact on many aspects of physical functioning. However, although each metrics was on average significantly different between groups and indicated large effect size, none showed a significant linear relationship (correlation) with the intensity of pain (VAS). The weakness of association demonstrates the heterogeneity of behaviors in response to pain and the difficulty to establish a single generic metric as objective outcome measure of physical functioning in chronic pain conditions.

Exploratory factor analysis revealed that the set of metrics clustered along two dimensions (factors), allowing to devise two composite scores, one integrating features of *activity/mobility*, and the other integrating features of *complexity*. These two scores were subsequently aggregated into a global composite that was negatively and significantly associated with the intensity of pain (when controlled for age and BMI). Although pain intensity accounted for a modest 25 % of variance in the global composite score, the suggestion is that only the assessment of the *overall* physical behavior has the potential to capture the impact of intensity of pain on daily life functioning. The observation that pain and levels of physical activity are related when it is based on broad/global assessment (and tend to disappear as the assessment become more specific) was first signaled three decades ago, although at that time the assessment of physical activity was based on self-report and observation [12]. Since then, studies that looked at correlations between pain intensity and measures of physical activity failed to provide consistent results [43, 44]. To the best of our knowledge, the present study is the first to *demonstrate* objectively that pain intensity is related significantly to the *overall* physical behavior of patients, and that global composite scores could be used as more sensitive outcome measures in clinical assessment and trials for treatment evaluation [4, 23].

As a study perspective, the preliminary results illustrated in Figs. 6 and 7 suggest that the multi-dimensional assessment of physical behavior using new paradigms such as pattern complexity might be useful in cluster analysis, to identify subgroups of patients, and to tailor the treatment according to the etiology (type) of chronic pain.

### Study limitations

This study was conducted retrospectively on data recorded in a heterogeneous sample of subjects in terms of pain severity and etiology, and demographics characteristics. The variance in physical behavior was possibly due to additional external sources, superposed to the presumed pain-related ones. However, the methodology could be used in future prospective studies using a similar set of metrics and additional information about the context of daily activities (employment status, professional work, etc.). The sample size was also relatively modest which may have led to under-powered analysis.

Another possible limitation is that the methods described involved data recorded with two body fixed movement monitors (trunk and thigh). However, the approach can be adapted for a simpler and more user-friendly monitoring setup, e.g. using a single inertial sensor fixed on sternum [26, 45].

## Conclusion

Chronic pain is a disabling experience affecting many aspects of functioning in everyday life. Clinical evidence indicates that the design of efficient treatment strategies, tailored to the patient, necessitates a reliable assessment of severity, impact, and type of pain. This study demonstrates that wearable technologies combined with appropriate analytical tools for data analysis and information extraction have the potential to provide an objective and comprehensive assessment of the impact of pain on domains of physical functioning in context of daily living.

## Abbreviations

CRPS: Chronic regional pain syndrome
ECDF: Empirical cumulative distribution functions
EFA: Exploratory factor analysis
FBSS: Failed back surgery syndrome
KS: Kolmogorov-Smirnov
SS: Spinal stenosis
VAS: Visual analogue scale
